# IgG4-Related Disease Presenting as Hypertrophic Pachymeningitis

**DOI:** 10.7759/cureus.21850

**Published:** 2022-02-02

**Authors:** Binita Sapkota, Ritesh Rampure, Murat Gokden, Sruthi Kanuru

**Affiliations:** 1 Rheumatology/Internal Medicine, University of Arkansas for Medical Sciences, Little Rock, USA; 2 Rheumatology, University of Arkansas for Medical Sciences, Little Rock, USA; 3 Pathology, University of Arkansas for Medical Sciences, Little Rock, USA

**Keywords:** igg4-related disease, immunosuppression, histopathology (hp), cns involvement, hypertrophic pachymeningitis

## Abstract

IgG4-related disease (IgG4-RD) is a multi-organ, immune-mediated inflammatory condition of unknown etiology characterized by infiltration of tissues by IgG4 producing plasma cells. IgG4-related disease (IgG4-RD) can ideally affect any organs, but the involvement of the central nervous system (CNS) is a rare entity. We present a case of a 67-year-old male who presented with diplopia with imaging showing hypertrophic pachymeningitis (HPM) and was diagnosed with IgG4-RD of the CNS based on histopathology report.

## Introduction

IgG4-related disease (IgG4-RD) of the central nervous system (CNS) is rare, and only about 82 cases have been described in literature who have hypertrophic pachymeningitis (HPM) as a manifestation of IgG4-RD [1]. The etiopathogenesis of IgG4-RD is unknown; however, it is believed that activated T and B cells stimulate fibroblasts, leading to collagen deposition and thickening of the affected tissues, e.g., the dura mater in the case of HPM [2]. Serum IgG4 level can be normal in patients with meningeal IgG4-related HPM, and although cerebrospinal fluid (CSF) IgG can be elevated, a meningeal biopsy is necessary to confirm the diagnosis and rule out other inflammatory, infectious, and neoplastic etiologies of hypertrophic pachymeningitis [3].

## Case presentation

A 67-year-old Caucasian male with medical conditions including transient ischemic attack, depression, polysubstance use, and ex-smoker presented with chief complaints of diplopia with looking straight and left lateral gaze of 10 days duration. The patient reported similar episode of diplopia one year prior to this hospitalization that resolved spontaneously within a day. Seven years prior to this presentation, he had whole left upper extremity numbness that lasted for 24 hours and resolved spontaneously. He denied any history of inflammatory eye disease, thrombosis, or risks for tuberculosis. He denied recent travel and unusual hobbies and did not have pets. He also denied any joint pain, joint swelling, fever, recent change in weight, skin rash, lymphadenopathy, oral ulcer, nasal ulcer, chest pain, shortness of breath, cough, hemoptysis, numbness, tingling, and muscle weakness.

His father had rheumatoid arthritis, and no family history of other autoimmune diseases or malignancy was reported. He had 40 pack-year smoking history and had quit seven years prior to this hospitalization. His home medications were aspirin, fluoxetine, and trazodone. His physical examination showed limitation of left eye abduction on left lateral gaze, consistent with left sixth nerve palsy, and there was no papilledema. Other examinations were within normal limits.

**Figure 1:**
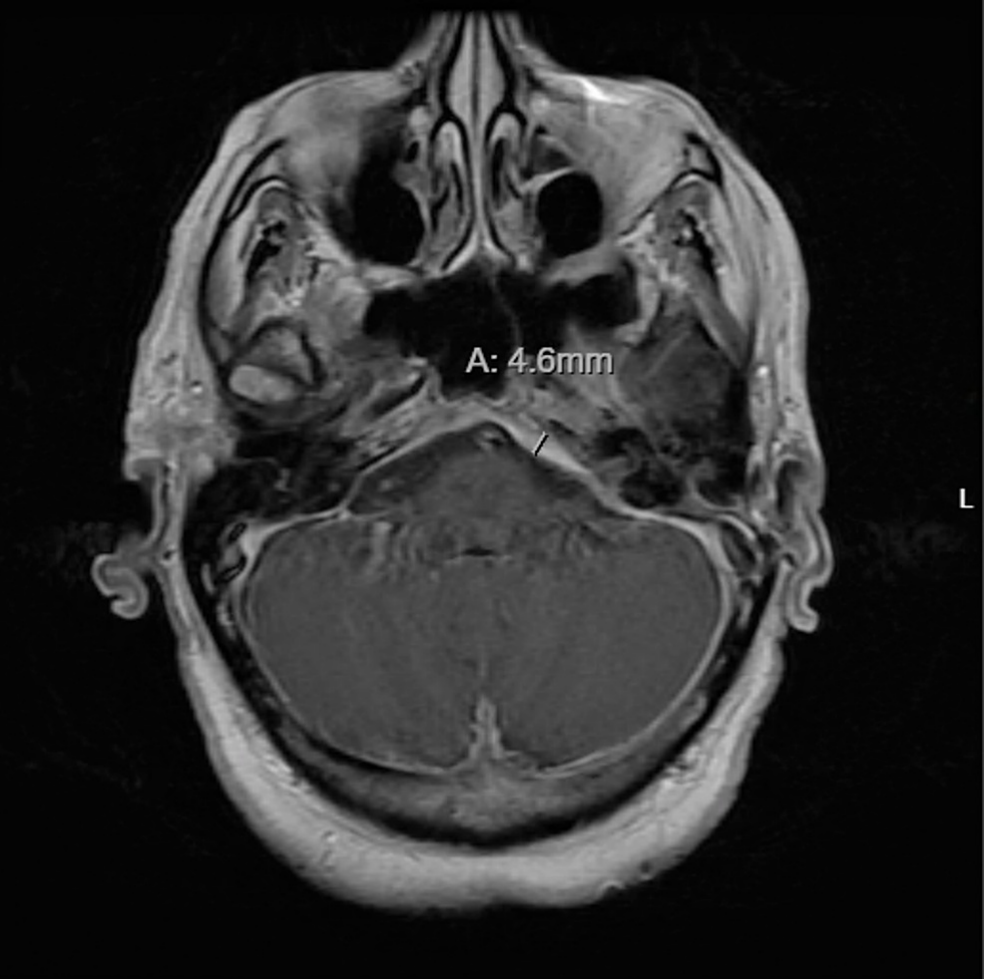
Brain MRI T1-weighted axial section showing thickening of the left clivus.

Laboratory results showed unremarkable cell counts and chemistry. Urinalysis did not show proteinuria or hematuria. Urine toxicology was positive for amphetamine and cannabinoids. Erythrocyte sedimentation rate was 44 mm/hour (reference range: 0-20 mm/hour), and C-reactive protein was 8.1 mg/dL (reference range: 0-5 mg/dL). Infectious workups including HIV, RPR, hepatitis B, hepatitis C, T-SPOT, HSV, VZV, and serum histoplasma antigen were negative. Autoimmune workups including antinuclear antibody, antineutrophil cytoplasmic antibody, rheumatoid factor, and angiotensin-1-converting enzyme were negative. C3 and C4 were within normal limits. CSF pressure was 13 cmH2O, and CSF analysis showed normal leukocyte count, protein, and glucose. CSF bacterial culture, AFB smear, fungal smear, VDRL, cryptococcus antigen, CMV, and EBV were negative. CSF IgG was 9.4 mg/dL (reference range: 0-6 mg/dL), and serum IgG and IgG4 were normal. Magnetic resonance imaging (MRI) of the brain with and without contrast showed pachymeningeal thickening and enhancement, including the posterior fossa, tentorium, internal auditory canals (IACs), left cavernous sinus, left Meckel's cave/trigeminal nerve foramen rotundum/ovale, left pterygopalatine fossa, and inferior orbital fissure extending the infraorbital nerve (Figure 1). With broad differentials in mind including infections, malignancies, and inflammatory diseases, e.g., granulomatosis with polyangiitis, polyarteritis nodosa, rheumatoid arthritis, and IgG4-related diseases, a meningeal biopsy was warranted for definitive diagnosis.

**Figure 2:**
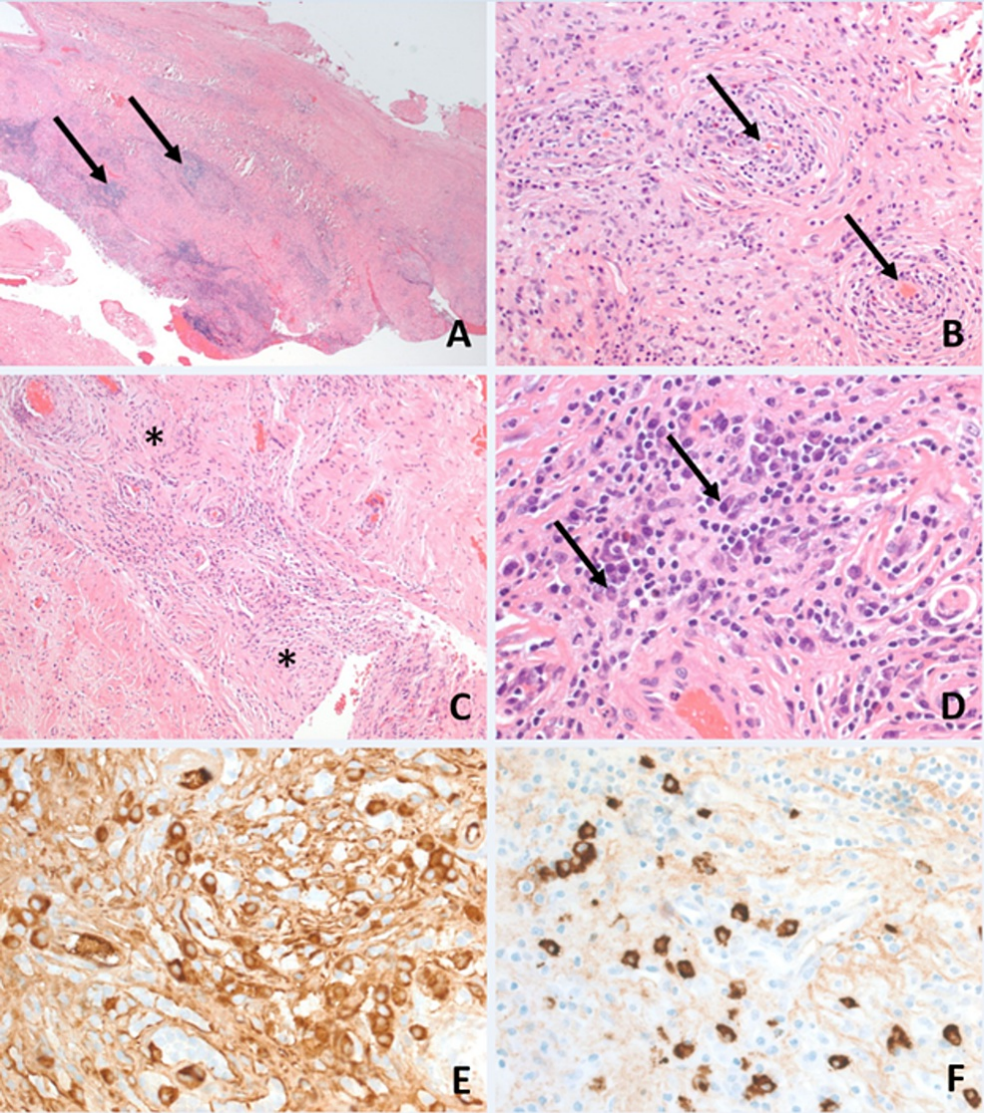
A: Prominent thickening of the meninges by fibrosis and inflammatory aggregates (arrows). B: Obliterative venulitis (arrows) with concentric thickening of their walls and narrowing of their lumina. C: Areas of storiform fibrosis (*) among the blood vessels. D: Lymphoid infiltrates are comprised mainly of small, round lymphocytes and plasma cells (arrows). E: Many IgG-positive plasma cells. F: Many IgG4-positive plasma cells. (A–D: H&E; E and F: immunohistochemistry; original magnifications: A, 40×; B, 200×; C, 100×; D and E, 400×).

Histopathology from meningeal biopsy revealed characteristic features of IgG4-related disease, including storiform fibrosis, IgG-positive cells, and IgG4-positive cells counted between 15 and 25 per one high-power field in various areas and constituting about 50% of the IgG-positive population, and obliterative venulitis. GMS and Ziehl-Neelsen stains were negative for fungal and acid-fast microorganisms (Figure 2).

He was started on prednisone 60 mg orally daily and had resolution of diplopia. The plan is to start rituximab for steroid-sparing effect and to prevent disease relapse.

## Discussion

IgG4-RD is an immune-mediated condition of unknown etiology characterized by infiltration of tissues by IgG4 producing plasma cells. A Japanese registry found the incidence of IgG4-RD to be about 0.28-1.08 per 100,000 population [4]. It can affect virtually any organ, although most commonly the major salivary glands, pancreas, lymph nodes, and tissues in the orbital and retroperitoneal spaces are affected. Umehara et al. proposed the following criteria for the diagnosis of IgG4-RD: (a) diffuse or localized swelling or masses in one or more organs; (b) elevated serum IgG4 levels (≥135 mg/dL); and (c) dense lymphoplasmacytic infiltration, storiform fibrosis, and/or obliterative phlebitis along with IgG4-positive plasma cells (>10-50 per high-power field depending on the organ or ratio of IgG4+/IgG+ of >40%) on histopathologic examination of the affected organs. The diagnosis is definite if all three criteria are met, probable if (a) and (c) are present, and possible if (a) and (b) are satisfied [4]. A serum IgG4 level elevated above 135 mg/dL has been found to have a sensitivity of 97% but poor specificity of 60% and a low positive predictive value of 34%. Up to 50% of patients with IgG4-RD could have normal serum IgG4 levels, giving it a poor diagnostic value [5].

CNS involvement in IgG4-RD is rare, mostly affecting the pituitary and meninges and rarely brain parenchyma. With meningeal involvement, there is focal or diffuse thickening of the dura mater, causing HPM in the brain or spinal cord or sometimes both. A review of cases of meningeal IgG4-RD showed that the median age affected with IgG4-related HPM was 53 years, and males were affected twice more than females, with the most common symptom being headache [6]. Clinical manifestations are either secondary to widespread meningeal involvement, which include headache, seizure, and cognitive impairment, or due to local compression causing cranial nerve palsies, cerebellar ataxia, or radiculopathies [7]. Cranial MRI demonstrates contrast-enhancing focal dural mass or diffuse dural thickening. A biopsy is necessary to confirm the diagnosis since neoplasia and inflammatory diseases, e.g., sarcoidosis, granulomatosis with polyangiitis, rheumatoid arthritis, and Sjogren's syndrome, can also present as HPM [8]. CSF analysis usually shows normal to mildly elevated protein, normal glucose, and variable lymphocytosis. There are reports of increased intrathecal IgG4 production. While CSF analysis by itself is not helpful diagnostically, elevated CSF IgG4 levels could provide an important diagnostic clue for IgG4-related HPM [9].

There is no definite treatment guideline for meningeal IgG4-RD. Systemic steroids are the first line of treatment. Treatment is often challenged with relapse in as high as 25%-50% of the cases after discontinuation or tapering of steroid [10]. There are studies demonstrating successful use of rituximab in IgG4-RD with clinical remission in up to 97% of cases even after stopping glucocorticoids within two months of treatment [11]. The use of rituximab as maintenance therapy has also been shown to prevent relapse [12]. Focal disease limited to a single organ, especially if associated with mechanical effects, can be treated with surgical excision.

## Conclusions

IgG4-RD affecting the CNS is rare, and it can affect the CNS in isolation or with other organs. We described a case of IgG4-related HPM presenting with diplopia. This case highlights that serum IgG4 levels can be normal in patients with IgG4-related HPM, and although CSF IgG can be elevated, a meningeal biopsy is necessary to confirm the diagnosis and rule out other inflammatory, infectious, and neoplastic etiologies of HPM. Although the exact pathogenesis of IgG4-RD is unknown, response to B cell depletion therapy indicates a role of B cells. Treatment should include the initial use of corticosteroids to prevent further damage, as well as the early institution of immunosuppressive therapy as a steroid-sparing agent, and also to prevent disease relapse.
